# Willingness and Predictors of Bystander CPR Intervention in the COVID-19 Pandemic: A Survey of Freshmen Enrolled in a Japanese University

**DOI:** 10.3390/ijerph192315770

**Published:** 2022-11-27

**Authors:** Yukihiro Mori, Yoko Iio, Yuka Aoyama, Hana Kozai, Mamoru Tanaka, Makoto Aoike, Hatsumi Kawamura, Manato Seguchi, Masato Tsurudome, Morihiro Ito

**Affiliations:** 1 Graduate School of Life and Health Sciences, Chubu University, 1200 Matsumoto-cho, Kasugai 487-8501, Aichi, Japan; 2 Center for Nursing Practicum Support, Chubu University, 1200 Matsumoto-cho, Kasugai 487-8501, Aichi, Japan; 3 Department of Lifelong Sports and Health Sciences, College of Life and Health Sciences, Chubu University, 1200 Matsumoto-cho, Kasugai 487-8501, Aichi, Japan; 4 Department of Clinical Engineering, College of Life and Health Sciences, Chubu University, 1200 Matsumoto-cho, Kasugai 487-8501, Aichi, Japan; 5 Department of Food and Nutritional Sciences, College of Bioscience and Biotechnology, Chubu University, 1200 Matsumoto-cho, Kasugai 487-8501, Aichi, Japan; 6 Department of Biomedical Sciences, College of Life and Health Sciences, Chubu University, 1200 Matsumoto-cho, Kasugai 487-8501, Aichi, Japan

**Keywords:** COVID-19 pandemic, bystander cardiopulmonary resuscitation, university freshmen students

## Abstract

The coronavirus disease 2019 (COVID-19) pandemic has decreased bystander cardiopulmonary resuscitation (BCPR) intervention rates. The purpose of this study was to elucidate the willingness of university freshmen to provide BCPR during the COVID-19 pandemic and the predictors thereof. A cross-sectional survey of 2789 newly enrolled university students was conducted after the end of the sixth wave of the COVID-19 epidemic in Japan; predictors of willingness to provide BCPR were assessed by regression analysis. Of the 2534 participants 1525 (60.2%) were willing to intervene and provide BCPR during the COVID-19 pandemic. Hesitancy due to the anxiety that CPR intervention might result in poor prognosis was a negative predictor of willingness. In contrast, anxiety about the possibility of infection during CPR intervention did not show a negative impact. On the other hand, interest in CPR and willingness to participate in a course, confidence in CPR skills, awareness of automated external defibrillation, and knowledge of CPR during the COVID-19 pandemic, were also positive predictors. This study suggests that the barrier to willingness to intervene with BCPR during a COVID-19 pandemic is not fear of infection, but rather hesitation due to the possibility of poor prognosis from the intervention. The significance of conducting this study during the COVID-19 epidemic is great, and there is an urgent need for measures to overcome hesitation regarding BCPR.

## 1. Introduction

In late 2019, severe acute respiratory syndrome coronavirus 2 (SARS-CoV-2) emerged, and the infectious disease caused by the virus, coronavirus disease 2019 (COVID-19), developed into a pandemic of global proportions [1]. As of September 2022, the virus has infected more than 610 million people and killed more than 6.5 million people worldwide. This situation has severely affected societies, economies, health systems, and human health worldwide.

Since the beginning of the pandemic, an increase in the incidence of out-of-hospital cardiac arrest (OHCA) has been reported [2,3]. Factors that increased OHCA incidence during the pandemic included behavioral limitations that reduced the likelihood that patients would present for acute care, including for care cardiac problems [4]. OHCA has a high mortality rate and remains one of the leading causes of death in developed countries [5,6]. It is well known that the outcome of OHCA depends on rapid intervention and that bystander cardiopulmonary resuscitation (BCPR) can improve survival [7]. However, since the beginning of the COVID-19 pandemic, it has been noted that bystanders were less willing to resuscitate OHCA patients in Canada [8]. A Japanese study also reported that OHCA patients, during the COVID-19 pandemic, were significantly more likely to not receive BCPR or automated external defibrillator (AED) treatment compared to those before the pandemic [9]. Emerging epidemics may negatively impact BCPR rates among OHCA patients due to fear of becoming infected [4,10,11,12].

In response to the spread of COVID-19, it is once again necessary to optimize the BCPR intervention rate and to specifically examine CPR education for bystanders. Therefore, the aim of this study was to elucidate the willingness to perform BCPR interventions during the COVID-19 pandemic and to clarify the factors associated with willingness among freshmen students at a Japanese university. We consider the students surveyed in this study as one of the important groups that can perform BCPR and disseminate CPR knowledge and skills.

## 2. Materials and Methods

### 2.1. Study Participants

The target population of this study was a group of 2789 new students that entered a single university in Aichi Prefecture, Japan, in April 2022. Only undergraduate students were included in the study. The university is a general university with many faculties, such as humanities, social science, natural science, and medical science. The survey was conducted in mid-April 2022. This was the time when the sixth COVID-19 epidemic in Japan was coming to an end. During the sixth epidemic, the number of daily infections in Japan exceeded 100,000 for the first time. At the time of the survey, there was no city blockade in Japan. In addition, the Japanese government had lifted priority measures, such as those to prevent the spread of the disease.

### 2.2. Survey

Data collection was performed anonymously using a questionnaire designed in Google Forms.

Participants responded with a Yes or No response as to whether they were willing to undertake BCPR interventions during the COVID-19 pandemic. They also responded to profile questions such as sex, enrolled faculty (medical science/others), and COVID-19-related questions, such as history of COVID-19 infection (personally and among individuals around them) and vaccination history. In addition, respondents were asked the following Yes/No questions: “During the COVID-19 pandemic, would you hesitate to perform CPR if you thought it might cause the patient to have a poor prognosis?” and “During the COVID-19 pandemic, would you be concerned about becoming infected during a BCPR intervention?” (Table 1).

In addition to the experience of actually encountering a CPR situation, the respondents were also asked about their experience, interest, and willingness to take CPR-related courses. Respondents were also asked to indicate their confidence in the following seven CPR-related skills by selecting “Yes” or “No”: “Checking the level of consciousness,” “Checking pulse and breathing,” “Chest compressions (cardiac massage),” “Airway clearance,” “Artificial respiration,” “Use of AED,” and “Handing over when the emergency services arrive” (Table 2).

The section on AEDs elicited responses regarding awareness of their intended use, experience of learning how to use them, and awareness of where they are located (Table 3).

We developed a 6-item question asking whether or not the respondents had knowledge of BCPR responses during the COVID-19 pandemic, referring to the guidelines (2020) of the Japan Emergency Medical Treatment Foundation’s Committee on Cardiopulmonary Resuscitation. The respondents were asked to answer each question (Table 4).

### 2.3. Statistical Analysis

The number and percentage of responses to all the questions were ascertained by descriptive statistics. The differences in willingness to intervene with BCPR during the COVID-19 pandemic (“Yes” or “No”) for each variable were then confirmed with a chi-square test. Next, logistic regression analysis (increasing variables method) was conducted to identify important predictors of willingness to intervene with BCPR during the COVID-19 pandemic. The dependent variable was willingness to intervene with BCPR during the COVID-19 pandemic (“Yes” = 1, “No” = 0). All explanatory variables were dummy variables with “Yes” = 1, “No” = 0. Adjusted odds ratios and 95% confidence intervals (95% CI) for each explanatory variable were calculated. The statistical significance level was *p* < 0.05. IBM SPSS Statistics version 27 (IBM Corp., Armonk, NY, USA) was used for statistical analysis.

## 3. Results

### 3.1. Study Participants

A total of 2552 respondents answered to the questionnaire, of which 18 were excluded for invalid responses or other reasons. As the result, 2534 respondents were included in the analysis; the mean age was 18.1 (±0.4) years. As shown in Table 1, 70.2% were male students and 14.7% were undergraduate students of medical science. Most of the respondents (91.0%) had a history of COVID-19 vaccination. On the other hand, 5.4% of the respondents had a history of COVID-19 while 33.8% had people (family members or acquaintances) with COVID-19 history.

### 3.2. Willingness to Intervene with BCPR during the COVID-19 Pandemic

Of the 2534 eligible participants, 1525 (60.2%) expressed willingness to intervene with BCPR during the COVID-19 pandemic. Males (*p <* 0.05) and students enrolled in medical science faculties (*p <* 0.01) were more likely to express a positive attitude towards their willingness to intervene with BCPR. Participants’ own history of COVID-19 were not associated with their intention to intervene in BCPR. Those who reported that someone around them had had the disease were more likely to report a willingness to intervene with BCPR (*p <* 0.01) (Table 1).

More than half of the respondents, 1468 (57.9%), expressed hesitancy to perform BCPR during the COVID-19 pandemic because it might lead to a poor prognosis for the recipient. A higher proportion of those who expressed this hesitancy were negative about their willingness to undertake BCPR interventions (*p <* 0.05). More than half (57.0%, 1445) of the respondents expressed concern that they would be infected if they were to conduct BCPR during the COVID-19 pandemic. Interestingly, a higher proportion of respondents in the group who expressed anxiety of infection was positive about their willingness to implement BCPR interventions *(p <* 0.01) (Table 1).

Only 2.8% had experience of actually encountering a CPR situation, 67.4% had attended a CPR course, 49.8% were interested in CPR, and 45.0% were willing to take a CPR course in the future. In the group that answered “Yes” to these questions, the proportion of those who were positive about their willingness to intervene with BCPR was generally higher (all *p* < 0.01) (Table 2).

Table 2 also shows the results of the survey on the confidence level for each CPR technique. The item with the highest percentage of respondents who felt confident was “checking the level of consciousness” (45.2%). The item with the highest proportion of respondents who were not confident was “artificial respiration” (86.6%). This was followed by “handover on arrival of the emergency services” (76.8%), and “chest compressions” (75.6%). The group that expressed confidence in each CPR skill had a higher proportion of respondents willing to intervene with BCPR for all seven items (all *p* < 0.01).

Table 3 shows that a high proportion of the respondents were aware of the purpose of AEDs (89.7%) and had learned how to use them (87.8%). However, the proportion of those who were aware of the location of AEDs (22.6%) was low. Those who answered “Yes” to these AED-related questions were more positive about their willingness to intervene with BCPR (*p* < 0.01).

Of the six items on knowledge regarding CPR response during the COVID-19 pandemic, the item with the highest percentage of knowledge was “Wash hands and face with soap and running water after handing over to emergency personnel” (38.4%). The second most common response was “For adult cardiac arrest, chest compressions and AED shocks should be administered without ventilation” (36.5%). Respondents who reported having knowledge were more positive in their willingness to intervene with BCPR for all six knowledge items (*p <* 0.01) (Table 4).

### 3.3. Predictors of Willingness to Intervene with BCPR

Table 5 shows the results of the analysis of predictors of willingness to intervene with BCPR during the COVID-19 pandemic: hesitancy that performing CPR might lead to a poor prognosis was highlighted as a factor negatively influencing willingness to intervene with BCPR (OR, 0.642 [95% CI, 0.526–0.785]; *p* < 0.01). In contrast, anxiety of infection during the BCPR intervention was found to be a rather positive factor (OR, 1.737 [95% CI, 1.431–2.109]; *p* < 0.01).

“Interest in CPR” (OR, 1.686 [95% CI, 1.364–2.084]; *p* < 0.01) and “willingness to take CPR courses in the future” (OR, 1.914 [95% CI, 1.546–2.369]; *p* < 0.01) were both shown to be positive factors for willingness to undertake BCPR interventions. Confidence in “checking pulse and breathing” (OR, 1.476 [95% CI, 1.180–1.848]; *p*< 0.01), “chest compressions” (OR, 1.780 [95% CI, 1.311–2.415]; *p* < 0.01), “airway clearance” (OR, 1.547 [95% CI, 1.159–2.065]; *p* < 0.01), and “handing over when emergency services arrive” (OR, 1.859 [95% CI, 1.394–2.480]; *p* < 0.01), were shown to be positive factors for willingness to perform BCPR interventions. “I know what an AED is for” (OR, 1.416 [95% CI, 1.033–1.941]; *p* < 0.05), “I have learned how to use an AED” (OR, 1.997 [95% CI, 1.492–2.672]; *p* < 0.01), and “I am usually aware of where AEDs are located” (OR, 2.511 [95% CI, 1.934–3.259]; *p* < 0.01) were also found to be positive factors for willingness to perform BCPR interventions.

Finally, knowledge that “for adult cardiac arrest, chest compressions and AED electroshock are administered without ventilation.” was suggested to be a positive predictor of willingness to perform BCPR interventions (OR, 1.585 [95% CI, 1.289–1.949]; *p* < 0.01). 

## 4. Discussion

This study examined the willingness of 2534 freshmen enrolled in a Japanese university during the COVID-19 pandemic to participate in BCPR interventions and the predictors thereof. We found that approximately 60% of respondents were positive about their willingness to undertake BCPR interventions during the COVID-19 pandemic. Notably, we found that hesitancy due to the anxiety that CPR interventions might have a poor prognosis is a factor negatively influences willingness to undertake the intervention. Interestingly, anxiety about the possibility of infection during CPR intervention did not negatively affect willingness to intervene with BCPR. Other positive predictors of willingness to intervene with BCPR included interest in CPR, willingness to participate in training courses, confidence in CPR skills, and awareness of AEDs. The study also suggested that having knowledge of CPR, including the precautions to take during the COVID-19 pandemic, was a significant predictor. 

A Taiwanese study of the general public of all ages during the COVID-19 pandemic reported that 39.0% of 1347 respondents had a positive attitude toward implementing BCPR [13]. Although the characteristics of the target population were different, our study population had a higher percentage of positive willingness to intervene compared to that of the study from Taiwan. This study investigated willingness to perform BCPR interventions rather than actual rates of performing CPR in the field. It has been suggested that the age of OHCA patients and their relationship with bystanders can influence their willingness to intervene [8]. However, our study did not limit the demographics of OHCA patients.

Even before the COVID-19 pandemic, fear of doing harm was cited as one of the reasons why bystanders did not offer CPR [14]. In the current study, conducted during the COVID-19 pandemic, it was also observed that hesitancy due to the possibility of poor outcomes was observed to negatively affects willingness to perform CPR. On the other hand, it was revealed that fear of infection during the COVID-19 pandemic may not necessarily have a negative impact on willingness to intervene with BCPR. Prior studies in Canada have indicated that fear of infection was a major obstacle to initiating BCPR even before the COVID-19 pandemic [8]. From the very beginning of the pandemic, an increased incidence of OHCA has been noted [2,15]. The probability of recovery from OHCA is low, and one factor that has been noted is the low rate of BCPR provision for OHCA due to fear of infection [4,10,11,12]. In this study, a higher proportion of the group with the knowledge that chest compressions and AED electroshock without ventilation could be administered for cardiac arrest in adults were more willing to perform BCPR. This knowledge component included exemption from ventilation with risk of infection. Originally, Japanese university students were reported to have relatively high knowledge, attitudes, and practice of COVID-19 infection control [16]. Anxiety about infection is likely to be an emotion that everyone has, even if they are positive about their willingness to participate in BCPR interventions. Further teaching and provision of solid information to bystanders with infection apprehension may lead to more positive changes in willingness to perform BCPR as the school year progresses.

Prior studies have reported inconsistent results regarding the percentage of willingness to perform BCPR interventions prior to the COVID-19 epidemic. For example, a survey of 4223 high school students, teachers, paramedics, nurses, and medical students in Japan before the COVID-19 pandemic indicated that 70–100% were willing to perform chest compressions on a stranger [17]. Furthermore, in a survey of Chinese students, also conducted before the COVID-19 pandemic, 59.7% were willing to perform BCPR on a stranger [18]. Unfortunately, however, we could not find any data examining the willingness of college freshmen to perform BCPR interventions before the COVID-19 pandemic. Therefore, since we have no data which can be compared with our present results, it needs further validation whether the results of our study are influenced by the pandemic.

In this study, interest in CPR and willingness to attend training were associated with willingness to undertake BCPR interventions; even before the start of the COVID-19 pandemic, areas with higher rates of BCPR implementation were reported to have a higher proportion of residents trained in CPR [19,20,21]. In addition, public health interventions, such as CPR and AED training programs for bystanders, are thought to be associated with an increased likelihood of performing BCPR and increased survival to discharge [22]. Therefore, the results of this study suggest that even during the COVID-19 pandemic, university students need to be motivated to be interested in CPR and to take CPR courses. As for the AED, it is an essential item that is key to successful CPR. In this study, we found for the first time that awareness of the purpose of AED use, their location, and learning experience, were strongly associated with willingness to intervene with BCPR during the COVID-19 pandemic. Since low rates of AED intervention by bystanders had been noted even before the COVID-19 pandemic [23], there is a need for additional AED training programs in educational institutions, such as schools [23]. However, the COVID-19 outbreak has also affected public participation in OHCA resuscitation in many countries, forcing citizen-oriented programs to either cease activity altogether or continue with restrictions [24]. However, the effectiveness of online-based CPR training has been shown previously [25]. In recent years, there have also been more opportunities for digital lectures in universities because of COVID-19, and this technology needs to be used effectively.

Our study shows that confidence in CPR skills, such as “chest compressions” and “airway clearance”, is associated with willingness to perform BCPR interventions. Since lack of confidence in skills is cited as a reason that bystanders do not provide CPR [14,26], this barrier needs to be overcome. Furthermore, in this study, in addition to these basic CPR skills, confidence in “checking pulse and breathing” was identified as a factor leading to willingness to perform BCPR. Studies reviewing the experiences of actual bystanders have identified sources of hesitation, such as uncertainty about whether a case is a cardiac arrest, as a factor that reduces motivation for BCPR interventions [27]. Therefore, for bystanders, pulse and breath confirmation skills are predicted to be an essential component of the decision to initiate CPR. In addition, confidence in “handing over on arrival of the emergency services” was found to be associated with willingness to intervene with BCPR. These procedural issues may also contribute to raise a decreased willingness to intervene with BCPR [14]. 

Our cross-sectional study was conducted at a single university after the sixth COVID-19 epidemic in Japan. The results may vary depending on the status of COVID-19 expansion and measures, such as behavioral restrictions issued by the government and local governments. In addition, the results may not be representative of the general population because of differences in CPR awareness, culture, lifestyle, and social conditions in different countries and regions. Furthermore, since differences exist in the ratio of male to female participants in this study, careful attention should be paid to this point. In addition, although there were approximately equal numbers of people interested in CPR and those not interested in CPR in this survey, the possible existence of selection bias due to the presence of those who declined to participate should also be considered. Finally, factors related to willingness to implement BCPR may exist beyond those identified in this study. We must continue to examine this from a broader perspective. However, the strength of this study is that the survey was conducted at a single, but comprehensive, university with faculties in multiple fields harboring students came from all over Japan. The results we present may be useful in designing educational programs within universities to improve BCPR implementation rates in response to the spread of COVID-19.

## 5. Conclusions

In this study, we elucidated the willingness of university freshmen to intervene with BCPR during the COVID-19 pandemic, and the barriers and facilitators thereof. We surmise that the barrier to willingness to participate in BCPR during the COVID-19 pandemic is not necessarily fear of infection, but hesitation due to the possibility of a poor prognosis resulting from the intervention. To overcome this problem, it is essential to promote interest in CPR and to provide solid knowledge and skills in CPR and AEDs even during the COVID-19 pandemic, even in university education. Therefore, we conclude that it is necessary to ensure opportunities for CPR education during the COVID-19 pandemic and to devise methods for its implementation.

## Figures and Tables

**Table 1:** Participant characteristics and their association with willingness to participate in BCPR interventions during the COVID-19 pandemic.

	Willingness to Intervene with BCPR				*p*-Value
	Yes (n = 1525, 60.2%)		No (n = 1009, 39.8%)		
	n	%	n	%	
Gender					
Male (n = 1779, 70.2%)	1098	61.7	681	38.3	<0.05
Female (n = 755, 29.8%)	427	56.6	328	43.4	
Undergraduate department (course, program, etc.)					
Medical science (n = 372, 14.7%)	259	69.6	113	30.4	<0.01
Others (n = 2162, 85.3%)	1266	58.6	896	41.4	
History of COVID-19 (respondent)					
Yes (n = 137, 5.4%)	84	61.3	53	38.7	0.781
No (n = 2397, 94.6%)	1441	60.1	956	39.9	
History of COVID-19 (family or acquaintances)					
Yes (n = 856, 33.8%)	546	63.8	310	36.2	<0.01
No (n = 1678, 66.2%)	979	41.7	699	58.3	
Previous vaccination with COVID-19 vaccine					
Yes (n = 2306, 91.0%)	1387	60.1	919	39.9	0.911
No (n = 228, 9.0%)	138	60.5	90	39.5	
During the COVID-19 pandemic, would you hesitate to perform CPR if you thought it might cause the patient to have a poor prognosis?					
Yes (n = 1468, 57.9%)	859	58.5	609	41.5	<0.05
No (n = 1066, 42.1%)	666	62.5	400	37.5	
During the COVID-19 pandemic, would you be concerned about becoming infected during a BCPR intervention?					
Yes (n = 1445, 57.0%)	971	67.2	474	32.8	<0.01
No (n = 1089, 43.0%)	554	50.9	535	49.1	

The *p*-values were calculated by the chi-square test. COVID-19, Coronavirus disease 2019; CPR, Cardiopulmonary resuscitation; BCPR, Bystander cardiopulmonary resuscitation.

**Table 2:** Associations between experience with CPR events, taking courses, interest, willingness to take courses, confidence in techniques, and willingness to intervene with BCPR during the COVID-19 pandemic.

		Willingness to Intervene with BCPR				*p*-Value
		Yes (n= 1525, 60.2%)		No (n = 1009, 39.8%)		
		n	%	n	%	
Experience of CPR situations, taking courses, interest, and willingness to take courses.						
Experience of actually encountering a CPR situation	Yes (n = 70, 2.8%)	57	81.4	13	18.6	<0.01
	No (n = 2464, 97.2%)	1468	59.6	996	40.4	
Previous CPR courses	Yes (n = 1709, 67.4%)	1118	65.4	591	34.6	<0.01
	No (n = 825,32.6%)	407	49.3	418	50.7	
Interest in CPR	Yes (n = 1262, 49.8%)	927	73.5	335	26.5	<0.01
	No (n = 1272, 50.2%)	598	47.0	674	53.0	
Willingness to take CPR courses in the future	Yes (n = 1140, 45.0%)	826	72.5	314	27.5	<0.01
	No (n = 1394, 55.0%)	699	50.1	695	49.9	
Confidence in CPR techniques						
Checking the level of consciousness	Yes (n = 1145, 45.2%)	821	71.7	324	28.3	<0.01
	No (n = 1389, 54.8%)	704	50.7	685	49.3	
Checking pulse and respiration	Yes (n = 919, 36.3%)	701	76.3	218	23.7	<0.01
	No (n = 1615, 63.7%)	824	51.0	791	49.0	
Chest compressions (Cardiac massage)	Yes (n = 618, 24.4%)	523	84.6	95	15.4	<0.01
	No (n = 1916, 75.6%)	1002	52.3	914	47.7	
Airway clearance	Yes (n = 644, 25.4%)	532	82.6	112	17.4	<0.01
	No (n = 1890, 74.6%)	993	52.5	897	47.5	
Artificial respiration	Yes (n = 339, 13.4%)	295	87.0	44	13.0	<0.01
	No (n = 2195, 86.6%)	1230	56.0	965	44.0	
Use of AEDs	Yes (n = 707, 27.9%)	585	82.7	122	17.3	<0.01
	No (n = 1827, 72.1%)	940	51.5	887	48.5	
Handover on arrival of emergency services	Yes (n = 589, 23.2%)	496	84.2	93	15.8	<0.01
	No (n = 1945, 76.8%)	1029	52.9	916	47.1	

The *p*-values were calculated by the chi-square test. COVID-19, Coronavirus disease 2019; CPR, Cardiopulmonary resuscitation; BCPR, Bystander cardiopulmonary resuscitation; AED, Automated external defibrillator.

**Table 3:** Relationship between response results on AEDs and willingness to intervene with BCPR during the COVID-19 pandemic.

	Willingness to Intervene with BCPR				*p*-Value
	Yes (n = 1525, 60.2%)		No (n = 1009, 39.8%)		
	n	%	n	%	
Know the purpose of an AED					
Yes (n = 2273, 89.7%)	1434	63.1	839	36.9	<0.01
No (n = 261,10.3%)	91	34.9	170	65.1	
Have experience learning how to use an AED					
Yes (n = 2226, 87.8%)	1411	63.4	815	36.6	<0.01
No (n = 308, 12.2%)	114	37.0	194	63.0	
I am usually aware of where AEDs are located.					
Yes (n = 573, 22.6%)	474	82.7	99	17.3	<0.01
No (n = 1961, 77.4%)	1051	53.6	910	46.4	

The *p*-values were calculated by the chi-square test. COVID-19, coronavirus disease 2019; BCPR, Bystander cardiopulmonary resuscitation; AED, Automated external defibrillator.

**Table 4:** Knowledge of CPR response during the COVID-19 pandemic and its association with willingness to intervene with BCPR.

	Willingness to Intervene with BCPR				*p*-Value
	Yes (n = 1525, 60.2%)		No (n = 1009, 39.8%)		
	n	%	n	%	
Knowledge A					
Yes (n = 718, 28.3%)	547	76.2	171	23.8	<0.01
No (n = 1816, 71.7%)	978	53.9	838	46.1	
Knowledge B					
Yes (n = 926, 36.5%)	709	76.6	217	23.4	<0.01
No (n = 1608, 63.5%)	816	50.7	792	49.3	
Knowledge C					
Yes (n = 706, 27.9%)	556	78.8	150	21.2	<0.01
No (n = 1828, 72.1%)	969	53.0	859	47.0	
Knowledge D					
Yes (n = 835, 33.0%)	619	74.1	216	25.9	<0.01
No (n = 1699, 67.0%)	906	53.3	793	46.7	
Knowledge E					
Yes (n = 682, 26.9%)	533	78.2	149	21.8	<0.01
No (n = 1852, 73.1%)	992	53.6	860	46.4	
Knowledge F					
Yes (n = 972, 38.4%)	723	74.4	249	25.6	<0.01
No (n = 1562, 61.6%)	802	51.3	760	48.7	

The *p*-values were calculated by the chi-square test. Knowledge A: Because cardiopulmonary resuscitation, including chest compressions alone, can produce aerosols, all cardiac arrest casualties should be treated as suspected infected during a COVID-19 outbreak. Knowledge B: For adult cardiac arrest, chest compressions and AED shocks should be administered without ventilation. Knowledge C: For cardiac arrest in children, if you have received training, have mastered ventilatory skills, and are willing to perform ventilation, artificial respiration should also be performed. Knowledge D: When checking a collapsed person’s reaction and breathing, do not get too close to the face. Knowledge E: If chest compressions are judged to be necessary after confirming respiration, perform chest compressions with a mask on if the casualty is wearing a mask to prevent aerosol dispersion, or with a towel or cloth over the casualty’s nose and mouth if they are not wearing a mask. Knowledge F: Wash hands and face with soap and running water after handing over to the first aid team. COVID-19, coronavirus disease 2019; BCPR, Bystander cardiopulmonary resuscitation; AED, Automated external defibrillator.

**Table 5:** Predictors of willingness to intervene with BCPR during the COVID-19 pandemic.

	OR	95% CI		*p*-Value
		Lower Limit	Upper Limit	
During the COVID-19 pandemic, I would hesitate to perform CPR because I thought that I might cause a poor prognosis.	0.642	0.526	0.785	<0.01
During the COVID-19 pandemic, I am concerned about being infected with the disease during a BCPR intervention.	1.737	1.431	2.109	<0.01
Interest in CPR	1.686	1.364	2.084	<0.01
Willingness to take CPR courses in the future	1.914	1.546	2.369	<0.01
Confidence in checking breathing and pulse	1.476	1.180	1.848	<0.01
Confidence to do chest compressions.	1.780	1.311	2.415	<0.01
Confidence in securing the airway	1.547	1.159	2.065	<0.01
Confident in handing over when the emergency services arrive	1.859	1.394	2.480	<0.01
Know the purpose of an AED	1.416	1.033	1.941	<0.05
Have experience of learning how to use an AED	1.997	1.492	2.672	<0.01
I am usually aware of where AEDs are located.	2.511	1.934	3.259	<0.01
Knowledge B ^†^	1.585	1.289	1.949	<0.01

The *p*-values were calculated by logistic regression analysis. The dependent variable was willingness to perform CPR during the COVID-19 pandemic (Yes = 1, No = 0). All explanatory variables were answered with Yes/No (Yes = 1, No = 0) (References were all “No”). OR, Odds ratio; CI, Confidence interval; COVID-19, Coronavirus disease 2019; CPR, Cardiopulmonary resuscitation; BCPR, Bystander cardiopulmonary resuscitation; AED, Automated external defibrillator; ^†^, For adult cardiac arrest, chest compressions and AED shocks should be administered without ventilation.

## Data Availability

Not applicable.
